# Transposable elements in phytopathogenic *Verticillium* spp.: insights into genome evolution and inter- and intra-specific diversification

**DOI:** 10.1186/1471-2164-13-314

**Published:** 2012-07-16

**Authors:** Stefan G Amyotte, Xiaoping Tan, Kayla Pennerman, Maria del Mar Jimenez-Gasco, Steven J Klosterman, Li-Jun Ma, Katherine F Dobinson, Paola Veronese

**Affiliations:** 1 University of Western Ontario, London, ON, Canada; 2 Department of Plant Pathology, North Carolina State University, Raleigh, NC, USA; 3 Department of Plant Pathology, Penn State University, University Park, PA, USA; 4 USDA-ARS, Salinas, CA, USA; 5 University of Massachusetts, Amherst, MA, USA; 6 Agriculture and Agri-Food Canada, London, ON, Canada

**Keywords:** Transposable elements, *Verticillium* spp., Retrotransposons, DNA transposons, Repeat-induced point mutation (RIP), TE domestication, Genome evolution

## Abstract

**Background:**

*Verticillium dahliae* (Vd) and *Verticillium albo-atrum* (Va) are cosmopolitan soil fungi causing very disruptive vascular diseases on a wide range of crop plants. To date, no sexual stage has been identified in either microorganism suggesting that somatic mutation is a major force in generating genetic diversity. Whole genome comparative analysis of the recently sequenced strains VdLs.17 and VaMs.102 revealed that non-random insertions of transposable elements (TEs) have contributed to the generation of four lineage-specific (LS) regions in VdLs.17.

**Results:**

We present here a detailed analysis of Class I retrotransposons and Class II “cut-and-paste” DNA elements detected in the sequenced *Verticillium* genomes. We report also of their distribution in other Vd and Va isolates from various geographic origins. In VdLs.17, we identified and characterized 56 complete retrotransposons of the Gypsy-, Copia- and LINE-like types, as well as 34 full-length elements of the “cut-and-paste” superfamilies Tc1/mariner, Activator and Mutator. While Copia and Tc1/mariner were present in multiple identical copies, Activator and Mutator sequences were highly divergent. Most elements comprised complete ORFs, had matching ESTs and showed active transcription in response to stress treatment. Noticeably, we found evidences of repeat-induced point mutation (RIP) only in some of the Gypsy retroelements. While Copia-, Gypsy- and Tc1/mariner-like transposons were prominent, a large variation in presence of the other types of mobile elements was detected in the other *Verticillium* spp. strains surveyed. In particular, neither complete nor defective “cut-and-paste” TEs were found in VaMs.102.

**Conclusions:**

Copia-, Gypsy- and Tc1/mariner-like transposons are the most wide-spread TEs in the phytopathogens *V. dahliae* and *V. albo-atrum*. In VdLs.17, we identified several retroelements and “cut-and-paste” transposons still potentially active. Some of these elements have undergone diversification and subsequent selective amplification after introgression into the fungal genome. Others, such as the ripped Copias, have been potentially acquired by horizontal transfer. The observed biased TE insertion in gene-rich regions within an individual genome (VdLs.17) and the “patchy” distribution among different strains point to the mobile elements as major generators of *Verticillium* intra- and inter-specific genomic variation.

## Background

Present in the environment as free-living organisms or in symbiosis with other organisms, fungi have a major impact on human society. Besides being major contributors to the decomposition and recycling of nutrients, they are largely employed in food production, biotechnologies and agricultural systems. However, these microorganisms also cause devastating diseases, particularly of plants, representing a major threat to global food security. It is noteworthy that a significant number of plant pathogenic fungi are able to cause serious human and animal diseases, for which there are very few effective therapeutic agents.

*Verticillium dahliae* (Vd) and *V. albo-atrum* (Va) (Eukaryota, Fungi, Ascomycota) cause very disruptive vascular diseases in over 400 plant species, including vegetable, ornamental and tree crops [1]. These microorganisms penetrate the root system of their hosts and induce typical leaf wilt symptoms by spreading throughout the xylem vessels and disrupting water transport. Compared to Va, Vd is generally characterized by a broader host range and geographical distribution, and the capability to produce longer lasting soil resting structures called microsclerotia. No sexual reproduction has been reported in either Vd or Va, thus implicating somatic mutation as a driving force in their evolution. Comparative analysis of the recently sequenced genomes of the isolates VdLs.17 (33.8 Mb) and VaMs.102 (32.8 Mb), showed that they are 97% identical and that VdLs.17 contains regions in chromosomes three and four that are not present in the Va isolate [2]. These lineage-specific (LS) regions, which encompass about 3.5% of the fungal genome, comprise about 350 protein-coding genes and are also enriched in repetitive sequences corresponding to both previously known [3,4] and as-yet uncharacterized transposon-like sequences [2].

Transposable elements (TEs) are found in virtually every prokaryotic and eukaryotic genome where they can exert a multifaceted mutagenic activity that leads to changes in chromosome architecture, generation of new regulatory networks and increases in the protein repertoire [5-7]. On the basis of structural-functional characteristics, TEs have been separated into two major classes each comprising subclasses or orders, superfamilies, families and subfamilies [8,9].

The elements of Class I, retrotransposons, replicate by a “copy-and-paste” mechanism that generates RNA intermediates, which are subsequently reverse transcribed into double-stranded (ds) DNA by TE-encoded enzymes. These retroelements have been recently classified into five orders comprised of long terminal repeat (LTR) elements, *Dictyostelium* intermediate repeat sequence (DIRSs), Penelope-like elements (PLEs), and long and short interspersed nuclear elements (LINEs and SINEs) [8,9]. Among the most widespread retrotransposons are the LTR superfamilies Gypsy and Copia, which include two ORFs coding for the structural virus-like protein GAG and the reverse transcriptase (RT)/integrase (INT) enzyme POL.

Class II elements, DNA transposons, are mobilized *via* the formation of DNA intermediates. If the intermediate is dsDNA, the transposition is called “conservative” and the elements are of the “cut-and-paste” type. Complete, *alias* autonomous, TEs of this type are generally delimited by terminal inverted repeats (TIRs) and encode a single transposase. The transposase binds conserved regions within the TIRs, mostly direct short tandem duplications, and catalyzes the excision of the complete element, which then reintegrates in a new genomic location. When TE mobilization proceeds *via* single-stranded (ss) DNA and does not involve the element excision it is defined as “replicative”, a mode of transposition that characterizes the more complex Helitron- and Maverick-like transposons [10,11].

Although fungal genomes generally contain fewer repetitive sequences than do higher eukaryotes [8,12], TEs have played critical roles in the evolution of their fungal hosts and, in particular, of the phytopathogens. In the rice blast fungus, *Magnaporthe oryzae*, for example, TEs were found to cluster in regions having high recombination rates and gene duplication events [13]. Similarly, in the black mold *Aspergillus niger*, specific retrotransposon-mediated recombinations have led to inversions of genomic regions [14]. In addition, host-specific toxin genes of *Cochliobolus carbonum*[15] and *Alternaria alternata*[16], as well as host-specificity genes of *M. oryzae*[17] are situated in transposon-rich regions of the genome. Because gene products conferring host-specificity can be recognized by plant host defense receptors, it has been hypothesized that this proximity could allow expansion of host range through TE-mediated gene loss [17]. In addition, TE distribution can be extremely variable between isolates of a single fungal species, and TEs have been used as markers to distinguish genetically divergent populations, and for the identification of subpopulations [18].

Introgression of mobile elements into the genome is potentially deleterious, and cells have evolved different processes for their elimination, including silencing by RNA interference and repeat-induced point (RIP) mutation [19-21]. The introduction of RIP mutations in the DNA of fungi is linked to sexual reproduction, occurring at the time of meiosis, and the process likely arose as a genome defense mechanism against the intrusion of repetitive DNA sequences. The RIP machinery recognizes duplicated sequences longer than 400 bp and sharing more than 80% identity, and introduces in both copies C:G to T:A transitions [22]. Noticeably, RIP has been detected in the asexual fungi *A. nigerPenicillium crysogenum* and *V. dahliae*[2,23], suggesting the existence of an ancestral or cryptic sexual state in these species. Interestingly, RIP mutations in VdLs.17 affected members of the Gypsy but not of the Copia superfamilies of retrotransposons, indicating either a differential susceptibility to RIP by different types of TEs, or introgression of already RIPed sequences from a sexual organism [2].

To further investigate the nature and extent of genetic diversity in *V. dahliae*, we have undertaken an analysis of TEs in this species. We report here the identification and characterization of Class I and II transposons in *V. dahliae*, an analysis of their distribution in *V. dahliae* and *V. albo-atrum*, and discuss their potential role(s) as generators of diversity in the *Verticillium* genomes.

## Results

### Transposable elements in *Verticillium dahliae* genome (strain VdLs.17)

**Figure 1:**
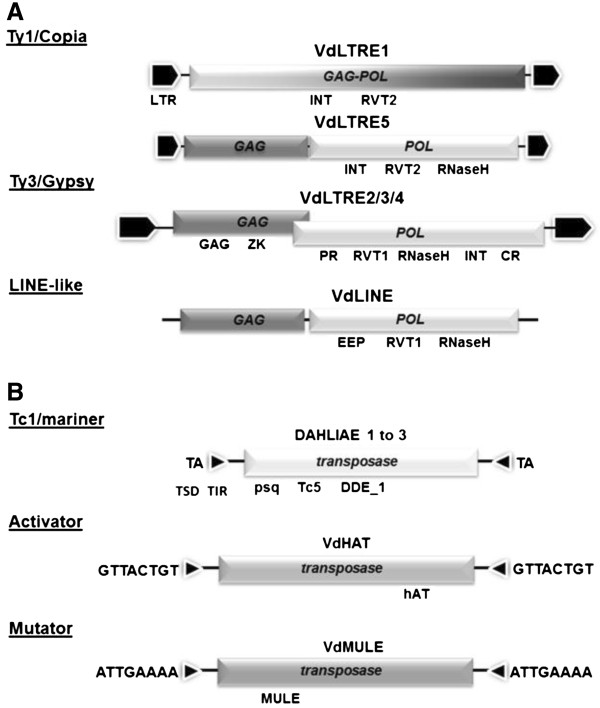
**Structure and organization of Class I and II elements identified in the genome of* Verticillium dahliae *(strain VdLs.17).A**) Class I LTR retrotransposons. Schematic representations of the structure of the Ty1/Copia-like and Ty3/Gypsy-like LTR elements, as well as a LINE-like non-LTR retrotransposon *.* The conserved domains identified in the putative protein sequences encoded by the *GAG* and *POL* genes are indicated below the open reading frames (ORFs). VdLTRE1 (upper schematic) contains direct long terminal repeats (LTR), and a fused *GAG* and *POL* ORF, while VdLTRE5 *GAG* and *POL* ORFs are separated by a UGA stop codon. The highly similar sequences of VdLTRE2, VdLTRE3, and VdLTRE4 (VdLTRE2/3/4) have overlapping *GAG* and *POL* ORFs. VdLINE sequence contains linked, but separate, *GAG* and *POL* ORFs. Domain abbreviations: INT, integrase (pfam00665); RVT2, reverse transcriptase 2 (pfam07727); RnaseH (pfam0075.16), GAG, group specific antigen; ZK, zinc knuckle (pfam00098); PR, Retropepsin protease (CD00303); RVT_1, reverse transcriptase 1; CR, chromatin organization modifier (CD00024); EEP, endonuclease-exonuclease-phosphatase; **B**) Class II transposable elements of the superfamilies Tc1/mariner, Activator and Mutator. Each TE type possesses terminal inverted repeats (TIRs) of different length flanking a transposase gene (shown is the corresponding ORF). The Tc1/mariner transposases are characterized by the endonuclease superfamily motif DDE_1 (pfam03184) and the presence of the additional N-terminal DNA-binding domains helix-turn-helix_pipsqueak (here indicated as psq, pfam05225), and helix-turn-helix_Tnp_Tc5 (here indicated as Tc5, pfam3221). The relative position of the hAT dimerization (pfam05699) and the MULE (pfam10551) domains characterizing the Activator and Mutator transposases are also shown. Flanking the TIRs are the nucleotide sequences of the direct target site duplications (TSDs) generated in the fungal genome by the TE insertion.

Using a combination of bioinformatics predictions and manual inspections, we identified 56 complete Class I retrotransposons and 34 Class II “cut-and-paste” DNA transposons in the VdLs.17 genome. Based on comparison to consensus sequence structures [8], the retrotransposons were identified as members of the LTR class Copia and Gypsy superfamilies, and LINE superfamily I, and the DNA TEs as subclass I members of the TIR superfamilies Tc1/mariner, Activator (hAT) and Mutator (MULE) (Figure 1, Table 1).

**Table 1:** ***Verticillium dahliae *(VdLs.17) Class I and II transposable elements identified in this study**

**Name**	**Family**	**Length (bp)**	**LTR (bp)**	**TIR (bp)**	**TSD (bp)**	**Copy number**^**a**^	**Element domain organization**^**b**^	**Best BLAST hit**^**o**^	**GenBank assession**
Class I									
VdLTRE1	Copia	5873	213	-	-	24	INT (3.53e-14); RVT_2 (6.12e-55); RH_Ty1 (1.96e-40)	EEA22357, *Penicilium mameffei*, identities 429/1277 (34%)	HM852745
VdLTRE2	Gypsy	6935	476	-	-	14	GAG (2.3e-10); CCHC (3.36e-06); PR (9.23e-20): RVT_1 (2.93e-26); RH_Ty3 (3.09e-51):	AAG24792 (Cgnet), *Collectotrichum gloeosporiodes*, identities 673/1149 (59%)	HM852746
VdLTRE3	Gypsy	6828	433	-	-	4	see VdLTRE2	AAG24792 (Cgnet), *Collectotrichum gloeosporiodes*, identities 661/1101 (61%)	HM852747
VdLTRE4	Gypsy	6870	455	-	-	4	see VdLTRE2	AAG24792 (Cgnet), *Collectotrichum gloeosporiodes*, identities 661/1106 (61%)	HM852748
VdLTRE5	Copia	5555	159	-	-	2	INT (1.01e-06); RVT_2 (8.41e-60); RH_Ty1 (6.26e-37)	EEA24286, *Penicilium mameffei*, identities 621/1306 (48%)	HM852749
VdLINE1	LINE	4905	-	-	-	8	APE (5.68e-10); RVT_1 (3.59e-20); RH (7.53e-09)	EEA18490, *Penicilium mameffei*, identities 879/1269 (70%)	HM852750
Class II									
DAHLIAE1a	Tc1/mariner	1861	-	45	2	12	HTH_psq (3.16e-06); HTH_Tnp_Tc5 (7.75e-04); DDE_1 (1.35e-58)	EGU83431, *Fusarium oxysporum*, identities 317/551 (58%)	JN160806
DAHLIAE1b	Tc1/mariner	1862	-	41	2	4	HTH_Tnp_Tc5 (4.32e-03); DDE_1 (6.10e-58)	EGU83431, *Fusarium oxysporum*, identities 317/539 (59%)	JN160807
DAHLIAE1c	Tc1/mariner	1862	-	55	2	1	HTH_psq (1.37e-06); DDE_1 (1.72e-59)	EGU83431, *Fusarium oxysporum*, identities 322/546 (59%)	JN160816
DAHLIAE1d	Tc1/mariner	1862	-	53	2	9	HTH_psq (1.37e-07); DDE_1 (1.72e-58)	EGU83431, *Fusarium oxysporum*, identities 317/548 (58%)	JN160808
DAHLIAE1e	Tc1/mariner	1861	-	62	2	1	DDE_1 (2.42e-46)	EGU83431, *Fusarium oxysporum*, identities 222/363 (61%)	JN160817
DAHLIAE2	Tc1/mariner	1909	-	62	2	1	HTH_psq (3.31e-08); HTH_Tnp_Tc5 (2.12e-12); DDE_1 (2.95e-68)	ABG26270 (OPHIO2), *Ophiostoma ulmi*, identities 275/529 (52%)	JN160809
DAHLIAE3	Tc1/mariner	1949	-	102	2	1	HTH_Tnp_Tc5 (1.36e-06); DDE_1 (5.53e-28)	EED116641, *Talaromyces stipitatus*, identities 148/400 (37%)	JN160810
VdHAT1	Activator	3164	-	22	8	1	Dimer_Tnp_hAT (7.15e-19)	EAQ93808, *Chaetomium globosum*, identities 361/814 (44%)	JN160811
VdHAT3	Activator	2809	-	20	-	1	Dimer_Tnp_hAT (1.54e-14)	EED11981, *Talaromyces stipitatus*, identities 265/652 (40%)	JN160812
VdMULE1	Mutator	3613	-	86	8	1	MULE (3.86e-17)	EFZ03845, *Metarhizium anisopliae*, identities 385/569 (68%)	JN160813
VdMULE2	Mutator	3538	-	84	-	1	MULE (3.49e-21)	EFZ03845, *Metarhizium anisopliae*, identities 306/441 (69%)	JN160814
VdMULE3	Mutator	3406	-	73	-	1	MULE (3.61e-20)	EFZ03845, *Metarhizium anisopliae*, identities 372/548 (68%)	JN160815

### LTR retrotransposons of superfamily Copia

We identified 26 full-length Copia-like TEs, referred to as VdLTRE 1 and 5, respectively (Figure 1A, Table 1). The 24 copies of VdLTRE1 were 5,873 bp in length, flanked by 213 bp LTRs, and contained a single ORF with fused *GAG* and *POL* sequences predicted to encode a 1621 amino acid (aa) polyprotein. In contrast, the two copies of VdLTRE5 had shorter (159 bp) LTRs, and *GAG* and *POL* ORFs separated by a UGA stop codon. This rarely observed ORF organization requires a leaky stop codon for translation read-through of the *POL* ORF 3’ into the *GAG* ORF. A conserved CARYYA sequence has been shown to be important for the stop codon read-through [24], however a search of VdLTRE5 showed that it lacked this canonical sequence motif. A MOTIF search identified in both VdLTRE1 and VdLTRE5 an integrase core domain located N-terminally to a reverse transcriptase domain, placing both elements as members of the Ty1/Copia family of retroelements [25]. These retrotransposases shared the highest sequences similarities with hypothetical proteins of the fungal animal pathogen *Penicillium marneffei*. Finally, a genome-wide search was conducted that identified two VdLTRE1 solo-LTRs. Such sequences are commonly observed remnants of LTR retrotransposons, and likely arise from recombination between the direct repeat sequences that flank retroelements [26].

### LTR retrotransposons of superfamily Gypsy

Three families of Gypsy-like elements were discovered and referred to as VdLTRE2, VdLTRE3, and VdLTRE4 (hereafter indicated as VdLTRE2/3/4), and corresponded to VDf35 and VDf90, previously identified as *V. dahliae* retrotransposon-like sequences [4]. These elements were ~ 6.8 to 6.9 Kb in size, 82.5% identical at the nucleotide level, encoded nearly identical GAG and POL proteins, and possessed LTRs of 476, 433 and 455 bp, respectively (Figure 1A, Table 1). There were 22 full-length elements and 14 solo-LTR sequences in the VdLs.17 genome. The *GAG* proteins of these Gypsy-like elements contained both gag and zinc finger conserved domains. The *POL* ORFs were in a −1 frameshift orientation relative to that of the *GAG* ORF, and comprised aspartate protease (PR), reverse transcriptase (RT), RNaseH, chromodomain, and integrase (IN) domains in an organization typical of Ty3/Gypsy-like elements [8,27]. The top hit from a BLASTP search with the 1129 aa VdLTRE2/3/4 POL predicted proteins was the Cgret LTR element from the closely related cranberry fruit rot fungus *Colletotrichum gloeosporioides*[28].

### Non-LTR retrotransposons of superfamily I

A LINE-like non-LTR retrotransposon, VdLINE1, was identified as present in 8 copies. The 4,905 bp elements contained separate *GAG*-like and *POL* ORFs, and an NCBI conserved domain search identified endonuclease-exonuclease-phosphatase (EEP) and RT domains, as well as a C-terminal RNaseH domain that defines superfamily I elements [8]. The top hit from a BLASTP search of the predicted 1245 aa POL protein was that of a putative reverse transcriptase of *P. marneffei* (Figure 1A; Table 1).

### “Cut-and paste” transposons of superfamily Tc1/mariner

Among the DNA transposons found in the VdLs.17 genome, twenty seven full-length Tc1/mariner-like elements were identified by having their short, perfect inverted terminal repeats (TIRs) flanked by dinucleotide TA target site duplications (TSDs), and the presence between the TIRs of a single intronless ORF encoding a transposase characterized by the conserved endonuclease domain DDE_1 [18] and the N-terminal DNA binding domains HTH_psq and HTH_Tnp_Tc5 (Figure 1B, Table 1, aa sequences of the conserved domains in Additional file 1; Figure S1). On the basis of TIR, ORF and whole TE length and sequence identity, we distinguished three VdLs.17 Tc1/mariner families referred hereafter as DAHLIAE 1, 2 and 3.

Among the DAHLIAE1 sequences, we found 25 complete elements about 1.9 kb in length. These sequences were associated with 41 to 62 bp TIRs that showed conservation of the first four nucleotides ACGT-, and contained 15 to 17 bp internal direct repeats (DRs) (Additional file 2: Figure S2). We also detected about 10 additional defective DAHLIAE1-like elements, five of which lack both TIRs (not shown). All DAHLIAE1 predicted ORFs started at TE nucleotide position 100 and encoded 551 aa proteins with the DDE_1 endonuclease domain spanning aa positions 154–364 and one or both the N-terminal DNA-binding domains HTH_psq and HTH_Tnp_Tc5 at aa positions 7–50 and 57–120, respectively (Figure 1B, Table 1). BLASTP with DAHLIAE1 proteins revealed greatest sequence similarities (58–61% aa identity) with sequences from ascomycete fungi, in particular, with a predicted protein from the phytopathogen *F. oxysporum* (strain Fo5176) (Table 1).

**Figure 2:**
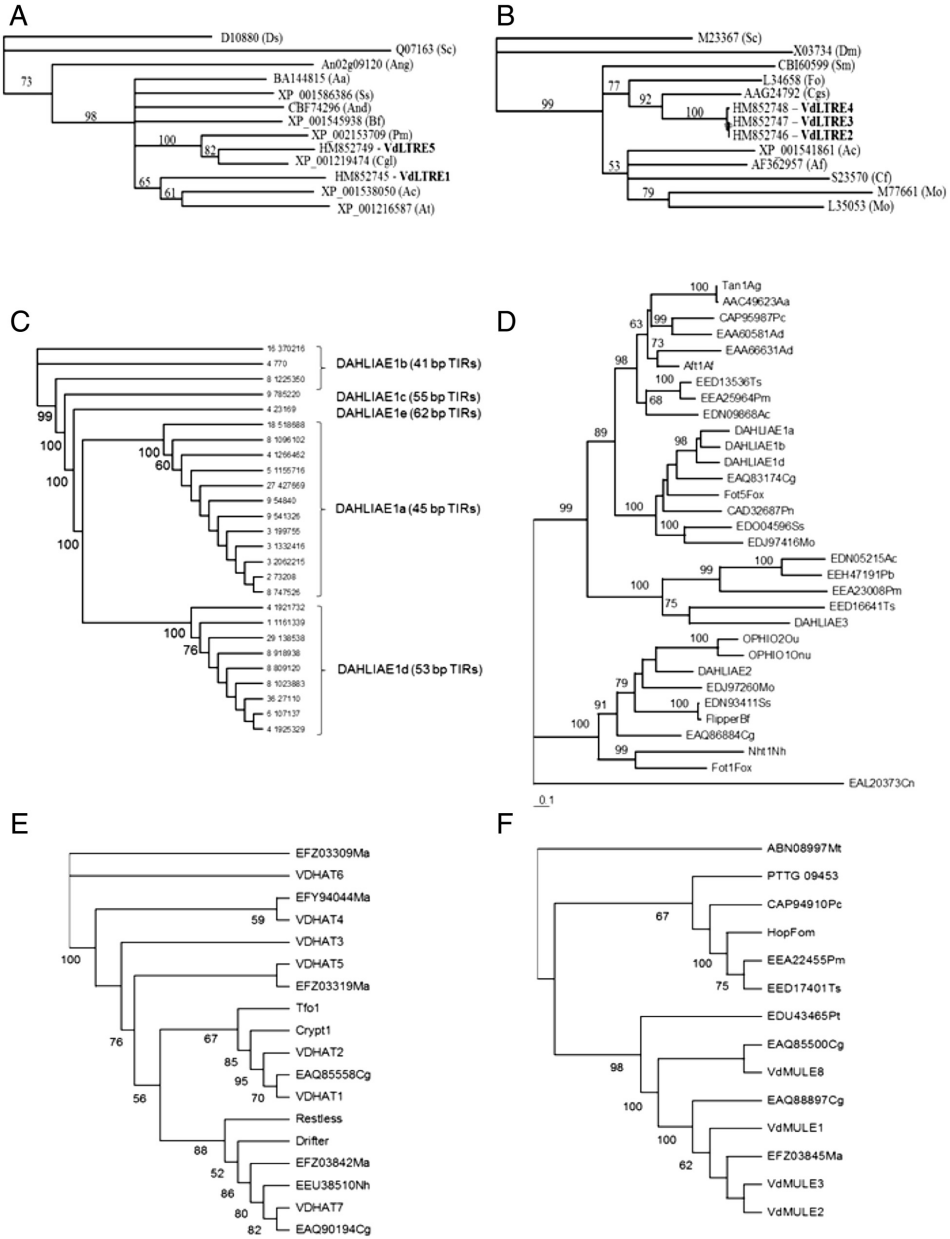
**Phylogenetic analysis of *VdLs.17* Class I and II elements. A**) Ty1/Copia LTR retrotransposons, analysis of the reverse transcriptases (amino acid sequences); **B**) Ty3/Gypsy LTR retrotransposons, analysis of the reverse transcriptases (amino acid sequences); **C**) Tc1/mariner-like DAHLAIE 1, analysis of the full-length elements (TIR-comprising nucleotide sequences). Each element is identified by its chromosome location (supercontig and nucleotide position); **D**) Tc1/mariner-like DAHLAIE 1 to 3, analysis of complete transposases (amino acid sequences); **E**) Activator-like VdHAT elements, analysis of hAT transposase domains (amino acid sequences); **F**) Mutator-like VdMULE elements, analysis of MULE transposase domains (amino acid sequences). The trees indicate the GenBank accession number of the amino acid sequences analyzed, along with the abbreviation of the scientific names of organisms of origin. Abbreviations are as follow: Aa, *Alternaria alternata* (A) *, Aspergillus awamori* (D) *; Ac, Ajellomyces capsulatus; Af, Aspergillus flavus* (A) *, Aspergillus fumigatus* (D) *; Ad/And, Aspergillus nidulans* (A) *, Ag/Ang, Aspergillus niger* (D) *; At, Aspergillus terreus; Bf, Botryotinia fuckeliana; Cn, Cryptococcus neoformans; Cgl/Cg, Chaetomium globosum; Cf, Cladospororium fulvum; Cgs, Colletotrichum gloeosporioides; Dm, Drosophila melanogaster; Ds, Drosophila simulans; Fo/Fox, Fusarium oxysporum; Fom, Fusarium oxysporum melonis; Ma, Metharhizium anisopliae; Mo, Magnaporthe oryzae; Mt, Medicago truncatula; Pc, Penicillium crysogenum; Pm, Penicillium marneffei; Pn, Phaesphaeria nodorum; Pt, Pyrenophora tritici-repentis; Sc, Saccharomyces cerevisiae; Ss, Sclerotinia sclerotiorum; Sm, Sordaria macrospora; Ts, Talaromyces stipitatus*. Scale bar corresponds to 0.1 substitutions per amino acid or nucleotide.

To establish the relationships among the DAHLIAE1 transposons, we performed phylogenetic analysis using the full-length nucleotide sequences, which resulted in the classification of the 5 subfamilies DAHLIAE1 a to e (Table 1, Figure 2C). Members of the same subfamily shared the same TIR length and were 95–100% identical to each other. Sequence homologies between members of the different subfamilies ranged from 73 to 74%. DAHLIAE 1a and 1d, with 12 and 9 complete copies each, respectively, were the largest subfamilies and the most abundant DNA transposons in VdLs.17 genome. DAHLIAE1 c and e were each present in only a single complete copy, and encoded what are likely to be nonfunctional transposases due to in-frame early stop codons.

Only one complete copy of DAHLIAE2 was detected in VdLs.17. The element was 1,909 bp long with 62 bp TIRs, and harbored an ORF coding for a putative 542 aa polypeptide (Table 1). The TIRs, like in DAHLIAE1, started with the nucleotides ACGT-, comprised three mismatches, and the DR sequence TTTCGGACACCCCCCCC- repeated three times at the 5’-end of the TEs (Additional file 2: Figure S2). The DAHLIAE2 ORF started at nucleotide position 187, and the encoded transposase shared the greatest overall sequence homology (52% aa identity) with OPHIO2 from the causal agent of Dutch elm disease *Ophiostoma ulmi*[29].

Like DAHLIAE2, only one full-length copy of DAHLIAE3 was present in VdLs.17. The consensus sequence was 1,949 bp long, with 102-bp TIRs. The 5’ TIR started with the sequence CCCGT- and TIR alignment revealed one mismatch, and lack of internal direct duplications or palindromes (not shown). DAHLAIE3 contained a 1,653-bp ORF starting at the TE nucleotide position 144, and encoded a 550 aa transposase with similarities to a putative 427 aa transposase from the fungal human pathogen *Talaromyces stipitatus* (Table 1).

### “Cut-and paste” transposons of superfamily Activator

Seven VdLs.17 sequences, designated VdHAT 1 to 7, were found to putatively encode 55 to 85 aa hAT dimerization domains, which characterize the carboxy termini of the transposases of Activator-like element transposases [30] (domain aa sequences shown in Additional file 3: Figure S3A). We were able to define the consensus sequence and flanking short imperfect TIRs of two single copy elements, VdHAT 1 and 3, and identify for VdHAT1 the 8-bp TSD GTTACTGT containing a typical central TA-dinucleotide. The complete VdHAT 1 and 3 elements were 3,164 and 2,809 bp in length, with 22 and 20 bp TIRs, respectively (Figure 1B, Table 1). The TIRs of each element comprised one mismatch and, while the VdHAT3 TIRs started with the sequence ATTTG-, VdHAT1 TIRs were characterized by the motif CAGNG- (Additional file 2: Figure S2), which is also present in other fungal hAT-like elements such as the *Cryphonectria parasitica* Crypt1 [31], the *F. oxysporum* Drifter and Tfo1 [32,33], and the *Tolypocladium inflatum* Restless [34]. Analysis of the VdHAT partial and complete nucleotide sequences using BLASTN and CLUSTALW revealed no significant regions of similarity to Activator-like sequences from other organisms, suggesting that each VdHAT was VdLs.17-specific. ORF Finder searches with VdHAT TEs and the analysis of corresponding ESTs supported the presence of a single transposase-coding gene. However, the presence in most of the VdHATs of in-frame early stop codons suggested that partial, likely non-functional proteins would be generated. BLASTX analysis showed that while the best hits for VdHAT 1 and 2 were two sequences from *C. globosum*, the best matches for VDHAT 3 to 7 sequences were to four different predicted proteins of the recently sequenced entomopathogenic ascomycete *Metarhizium anisopliae*[35] (Table 1, Additional file 4: Table S1).

### “Cut-and paste” transposons of superfamily Mutator

BLASTP search using the 94 aa MULE domain from the active Mutator-like element Hop1 of *F. oxysporum* f. sp. *melonis*[36] revealed the presence of eight predicted MULE proteins in VdLs.17 (domain aa sequences shown in Additional file 3: Figure S3B). Manual inspection of the corresponding genomic sequences, which we refer to as VdMULE 1 to 8, resulted in the discovery of three potentially complete elements VdMULE 1 (3.6 Kb), 2 (3.5 Kb) and 3 (3.4 Kb), each encoding a single ORF, and containing 86, 82, and 53 bp TIRs, respectively. An 8 bp TSD (ATTGAAAA) was associated with one element only, and defective elements ranged in size from 363 to 2,371 bp (Table 1, Additional file 4: Table S1).

Alignment of the VdMULE nucleotide sequences with Hop1 TIRs showed the presence of the terminal consensus GGNAA, and of sub-terminal DRs of different lengths. In particular, while VdMULE 1 and 2 had three 15-bp and 17-bp repeats, respectively, VdMULE3, like Hop1, possessed 5 and 6 bp duplications (Additional file 2: Figure S2). Among the VdMULEs, the highest nucleotide sequence homology was observed between the three full-length elements (55% to 69% identity). VdMULE8 appeared to be the most divergent, with no significant similarity to any other VdMULE element.

VdMULE elements were predicted to harbor single transposase genes with different exon/intron structures. In particular, we found a single exon in the VdMULE1, 2, 3 and 6 genes, four exons in VdMULE4, two exons in VdMULE5 and five exons in the VdMULE8 genes, respectively. Complete MULE domains (94–95 aa) were present only in transposases of the full-length elements. The remaining transposases were characterized by N- or C-terminal domain truncations of 21 to 57 aa. Notably, the predicted 489 aa VdMULE7 protein (VDAG_04885) was found to be the in-frame product of the fusion between a nitrilase and a MULE-transposase coding sequence. The hypothetical protein harbors a 177 aa N-terminal nitrilase domain (pfam00795; involved in nitrile hydrolysis to ammonia and carboxyl acid) spanning aa positions 3–180, and a truncated 74 aa MULE domain spanning aa positions 363–436. VdMULE8 transposase (VDAG_04851) architecture was also characterized by the presence of a 20 aa zinc-knuckle (zf-CCHC, pfam00098) at its carboxy terminus. Proteins with the highest sequence homology to the VdMULE transposases, which ranged from 55% to 79% identity, were from *C. globosum* and *M. anisopliae* (Table 1).

### Distribution of Class I and II TEs in the VdLS.17 genome

**Figure 3:**
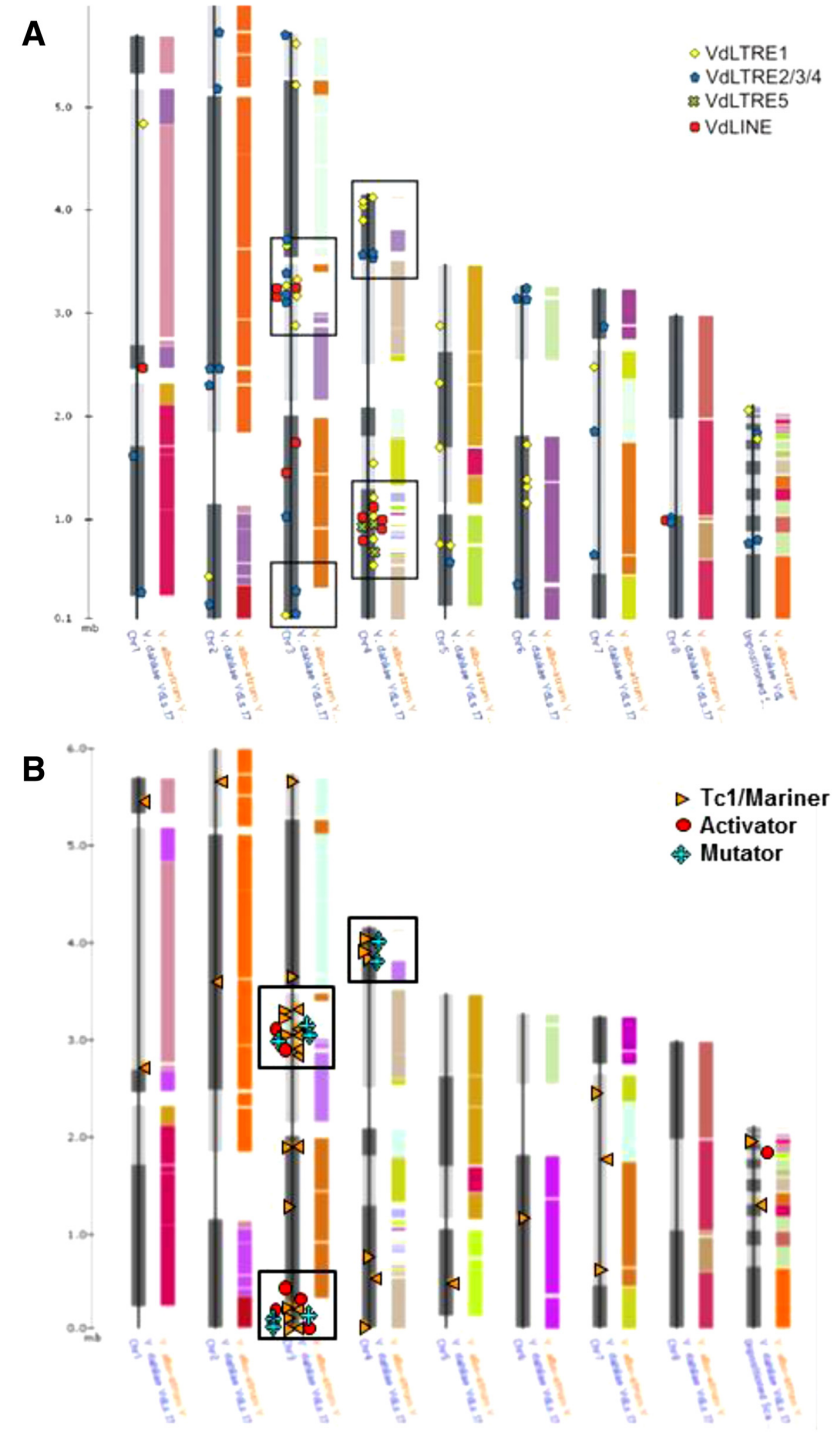
**Distribution of Class I and II TEs within the VdLs.17 genome.** The ChromoMap genome browser available online at the BROAD Institute website was used to visualize the TE sequences against a synteny map of the VdLs.17 and VaMs.102 sequenced genomes. The biased chromosome localization of the retroelements ( **A**) and the DNA elements ( **B**) in the “lineage-specific” regions of Vd.Ls17 are outlined in black.

**Figure 4:**
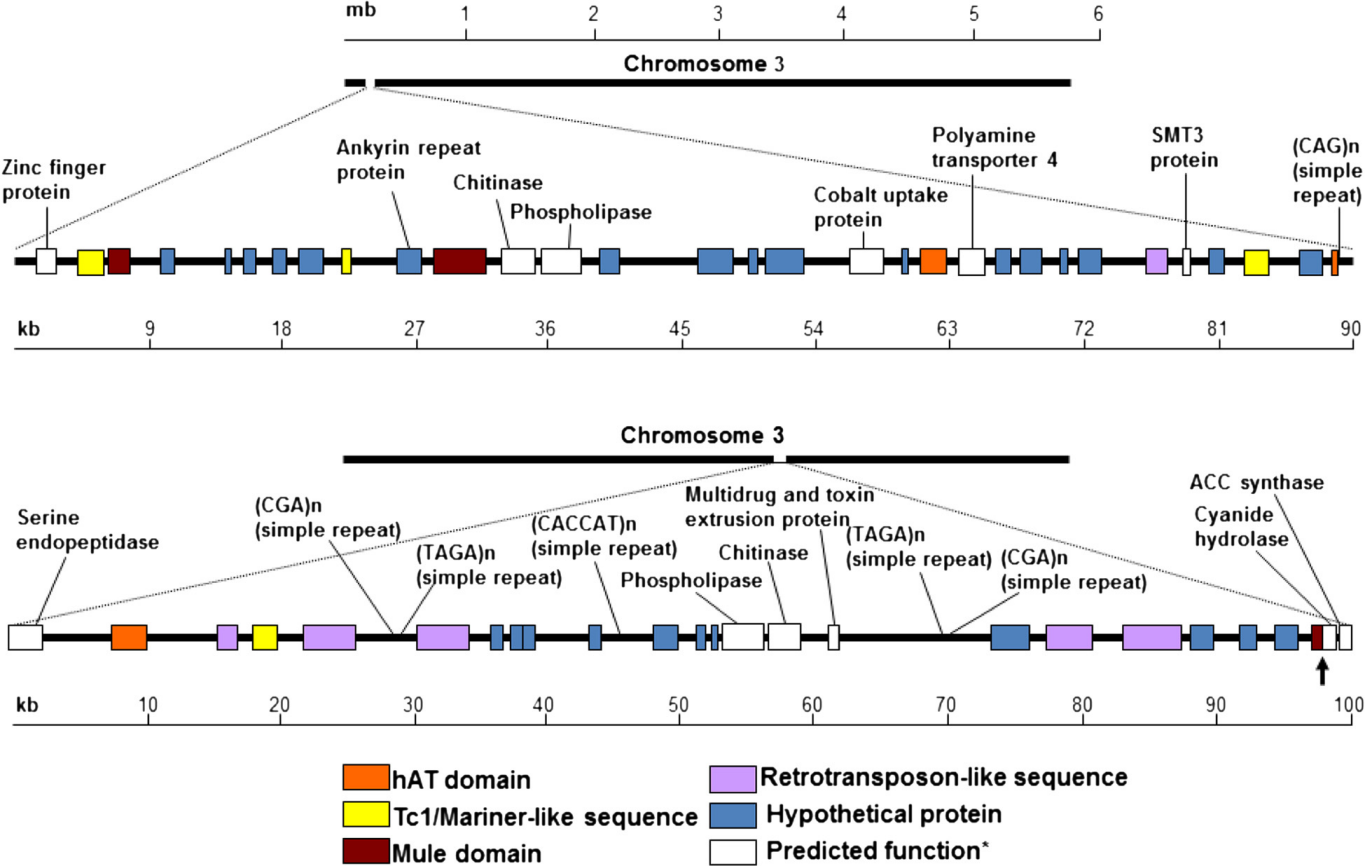
**Genetic context of Class I and II transposable elements in VdLs.17.** The sequence segments include 90 kb from supercontig 4 nucleotide positions 162,000-222,000 (top), and 100 kb from supercontig 8 nucleotide positions 1006000 – 1106000 (bottom), of chromosome 3. The arrow indicates the position of the putative cyanide hydratase fusion protein (VDAG_04885) containing both nitrilase and MULE transposase functional domains (pfam 00795 and 10551, respectively). *Predicted gene names were assigned by the Broad Institute (www.broadinstitute.org/annotation/genome/verticillium_dahliae).

An in-depth analysis of transposon distribution in VdLs.17 genome revealed that the most abundant elements, VdLTREs 1–4 and Tc1/mariner DAHLIAE1, were dispersed throughout the genome’s eight chromosomes, and in unpositioned scaffold sequences. However, there was notable TE clustering in chromosomes 3 and 4 (Figure 3 A and B). In particular, supercontings 4 and 8 of chromosome 3 appeared to contain “hot-spots” of TE insertions, harboring 21% and 20% of the transposons, respectively. These supercontigs also contain VdLs.17 LS (lineage-specific) regions 1 and 2, which are absent from the VaMs.102 genome [2]. The non-random TE distribution of the DDE_1, hAT and MULE motifs was further assessed, and odds ratio analyses [37] did indeed show that the LS regions are significantly enriched in transposon motifs (*P* < 0.05) relative to that of the total predicted gene distribution in the genome assembly of VdLs.17 (Additional file 5: Table S2). One approximately 100 Kb region from each of LS regions 1 and 2 was selected to illustrate the genetic context of some TEs (Figure 4). This figure highlights how the transposons are present in gene-rich locations, and shows a putative sequence duplication event involving genes encoding a chitinase and a phospholipase, in addition to those previously described [2].

### The *Verticillium albo-atrum* VaMs.102 genome contains few transposons

While TEs were prevalent in the *V. dahliae* VdLs.17 genome, few repetitive sequences were identified in VaMs.102. BLASTN and BLASTX queries against the *V. albo-atrum* genome using VdLs.17 Class I and II transposons identified one intact, full-length element having 99.9% sequence identity to VdLTRE1, one full-length but degenerate Copia-like element, and one partial sequence of approximately 1 kb, also of the Copia-type. Only short (45 to 263 bp) sequences were found that had 80 to 91 percent identity to the Gypsy-like elements. Lastly, no sequences corresponding to VdLTRE5 were identified, and remarkably, neither full-length nor incomplete Class II TEs were detected in the VaMs.102 genome.

### VdLs.17 Class I and II transposons are transcriptionally active

To assess potential transcriptional activity, we inspected the three VdLs.17 EST libraries generated in support of the *Verticillium* genome sequencing project [2] in search of sequences corresponding to the different TE types described herein. These libraries were obtained from fungal cultures grown on control complete medium (CM; ~10,000 ESTs), CM supplemented with root extract from the host plant lettuce (~5, 000 ESTs), or CM lacking nitrogen (~5, 000 ESTs) [2]. We identified ESTs matching all of the Class I and II transposons in at least one of the growth conditions (Table 2).

**Table 2:** Survey of VdLs.17 ESTs corresponding to the transposons identified in this study

**Transposon**	**Number of ESTs**^**a**^		
	**CM**	**CM + RE**	**CM - N**
Class I			
VdlTRE1	43	21	23
VdlTRE2	31	26	33
VdlTRE3	24**	16	21***
VdlTRE4	25**	23*	28
VdlTRE5	3	2	5
VdLINE1	13	11	4
Class II			
DAHLIAE1a	4	-	6
DAHLIAE1b	-	4	4
DAHLIAE1d	3	-	11
DAHLIAE1e	6	-	3
DAHLIAE2	6	2	5
DAHLIAE3	-	4	-
VdHAT1	12	2	8
VdHAT2	-	1	-
VdHAT3	-	1	-
VdMULE2	4	2	-
VdMULE3	-	5	-
VdMULE8	-	2	-

^a^VdLs.17 expressed sequence tag (EST) collections were generated from cultures grown in CM (complete medium), CM + RE (CM amended with root extract), or CM-N (CM lacking nitrogen source). For VdLTRE2, VdLTRE3 and VdLTRE4, the corresponding ESTs aligned with all three elements except that * one EST aligned with only VdLTRE4, ** four ESTs aligned with both VdLTRE 3 and 4, and *** one EST aligned with only VdLTRE3.

The EST analysis showed that Class I elements were transcriptionally active under all of the growth conditions. Although similar levels of expression were observed for the CM + root extract and CM -nitrogen cultures, numbers of VdLTRE1 ESTs were substantially higher than those of all other elements. In contrast, within each DNA TE family, a differential level of expression was observed according to the fungal growth condition. For instance, while DAHLIAE1 a, d and e were transcriptionally active primarily in control and nitrogen starvation conditions, ESTs for DAHLIAE3, VdHAT 2 and 3 and VdMULE 3 and 8 were detected only in the library from cultures grown in the root extract-amended medium.

Abiotic stress can stimulate expression of TEs in fungi [38,39]. We therefore used reverse transcription-PCR to make a semi-quantitative assessment of the expression of selected VdLs.17 DNA transposases in response to a heat shock treatment (42°C for 40 minutes). The resulting data provided evidence that while the expression of DAHLIAE1, DAHLIAE3, VdMULE2, and VdMULE3 was induced at 42°C, VdMULE8 expression appeared to be unaffected by the treatment, and DAHLIAE2 responded negatively to the treatment (Additional file 6: Figure S4).

### Phylogenetic analyses

To gain a better understanding of the evolutionary history of the different transposable elements identified in VsLs.17, we studied their phylogenetic relationships with homologous sequences from other fungal species. Analyses were done as described in the Methods section and using the aa sequences of the retroelement RT domains, predicted aa sequences of the complete Tc1/mariner proteins, and conserved domains of the VdHAT and VdMULE transposases (Figure 2).

Consistent with the predictions based on the organization of the POL domains, VdLTRE1 and VdLTRE5 clustered with known fungal Copia-like elements. However, bootstrap support separated the two types of elements into distinct clades, with VdLTRE1 being most closely related to sequences from *Aspergillus capsulatus* and *A. terreus* (Figure 2A). VdLTREs 2, 3 and 4 clustered together and with known Gypsy elements, showing close relationships with the *C. gloeosporioides* element Cgret [28] and Skippy from *F. oxysporum*[40] (Figure 2B).

Although bootstrap values supported a monophyletic origin for all the fungal DDE_1 transposases considered, none of the VdLs.17 DDE_1 transposases clustered with sequences from the basidiomycetes *Cryptococcus neoformans* (animal pathogen) and *Ustilago maydis* (plant pathogen), or with sequences from the four different *Aspergillus* species included in the analysis (Figure 2D). While the three DAHLIAE families, whose members share 28 to 34% identity, separated into 3 different lineages before speciation, DAHLIAE1 differentiated into subfamilies, sharing 67 to 72% aa identity, apparently after insertion into the VdLs.17 genome. Both DAHLIAE1- and DAHLIAE2-containing clades also comprised proteins from the Sordariomycetes *C. globosumF. oxysporumM. oryzae* and the Leotiomycete *S. sclerotiorum*, which are closely related to *Verticillium* spp. However, DAHLIAE 3 grouped with sequences from the more distantly related Erotiomycete species *Ajellomyces capsulatusP. marneffei, Paracoccidioides brasiliensis* and *T. stipitatus*. Among the fungal proteins clustering with DAHLIAE2, all of which possess the HTH_psq/HTH_Tnp_Tc5/DDE_1 domain organization, we found transposases of well characterized plant pathogen TEs such as Flipper of *B. fuckeliana*[41], Fot1 of *F. oxysporum*[42], Nht1 of *Nectria haematococca*[43], and OPHIO 1 and 2 of *O. nova-ulmi*[29].

Sequences with similarities to VdLs.17 hAT and MULE domains were identified in a much more limited number of fungi. Sequence alignments of the VdhAT and VdMULE domains revealed core regions of 53 and 69 aa, respectively, and led to the identification of amino acid positions that are conserved across all fungal species examined (alignments shown in Additional file 3: Figure S3). While the VdHATs were separated into 6 different clades, all VdMULEs clustered together (Figure 2 E and F). Bootstrap values in the range of 50–60% or below did not support a monophyletic origin for the VdHATs. In the clade containing VdHAT 1 and 2, there were sequences from the Activator-like transposons Crypt1 of the chestnut blight fungus *Cryphonectria parasitica*[31] and Tfo1 of *F. oxysporum*[33]*,* together with one sequence from *C. globosum*. VdHAT7 was more closely related to Drifter, a hAT element from *F. oxysporum*[32], and Restless from the cyclosporine-producing fungus *T. inflatum*[34], as well as to sequences from *C. globosumM. anisopliae*, and *N. haematococca*. VdHATs 3 to 6 were grouped with *M. anisopliae* sequences only (Figure 2E). The VdMULE clade comprised *M. anisopliae* and *C. globosum* sequences and appeared to be unrelated to the clade with the *F. oxysporum* element Hop and sequences from *P. crysogenum, P. marneffei*, and *T. stipitatus* (Figure 2F).

### Evidence of repeat induced point (RIP) mutation in VdLs.17 TEs

In a previous study we surveyed VdLs.17 retrotransponsons for evidence of RIP-like mutations and found them only in Gypsy-like sequences [2]. Here we extended the analysis to all VdLs.17 DNA transposons. Although the sequence divergence for the Activator- and Mutator-like elements was significant, RIPCAL searches [44], which included VdLTRE2-like sequences as positive control for RIPing, revealed no clear bias for RIP-like mutations in any of the VdLs.17 “cut-and-paste” elements (Additional file 7: Figure S5).

### Distribution of the VdLs.17 Class I and II TEs in other *V. dahliae* and *V. albo-atrum* isolates

The distribution of the Class I and II transposons identified in VdLs.17 was investigated in another 21 *V. dahliae* and *V. albo-atrum* isolates from various hosts and geographic regions, including the *V. dahliae* vegetative compatibility group (VCG) tester strains described by Joaquim and Rowe [45] (results summarized in Table 3 and shown in Additional file 8: Figure S6). The Tc1/mariner DAHLIAE2 showed the most widespread distribution among the two species, being present in 33% and 66% of the Vd and Va isolates, respectively. The Copia VdLTRE1 and the Gypsy VdLTR2/3/4 were detected in multiple copies in almost all Vd isolates, whereas the Copia-like element VdLTRE5 was found only in VdLs.17 and four other Vd isolates, and always in low copy number (Additional file 8: Figure S6A). Only four Va isolates were conclusively positive for the presence of VdLS.17-like retroelements. The Tc1/mariner DAHLIAE1 and Activator-like sequences were, like the retroelements, limited in their distribution, and represented primarily in *V. dahliae* (Additional file 8: Figure S6B). Lastly, Tc1/mariner DAHLIAE3 and VdMULE elements appeared to be specific to VdLs.17.

**Table 3:** **Survey of* Verticillium dahliae *and* V. albo-atrum *isolates for the presence of Class I and II transposons identified in VdLs.17 genome**

**Isolate**	**Geographic Origin**	**Host**	**VCG**^b^	**Retrotransposons**	**“Cut and Paste” DNA transposons**
*V.dahliae*					
VdLs.17^a^	CA	Lettuce	2B	Copia (VdLTRE 1 and 5), Gypsy (VdLTRE2/3/4)	Tc1/mariner (DAHLIAE 1 to 3), Activator (VdHAT 1 to 7), Mutator (VdMULE 1 to 8)
VdT9	CA	Cotton	1	Copia (VdLTRE 1 and 5), Gypsy (VdLTRE2/3/4)	-
VdV44	TX	Cotton	1	Copia (VdLTRE 1 and 5), Gypsy (VdLTRE2/3/4)	-
VdPCW	CA	Pepper	3	Copia (VdLTRE 1 and 5), Gypsy (VdLTRE2/3/4)	Tc1/mariner (DAHLIAE1d)
Vd70.21	unknown	unknown	3	Copia (VdLTRE 1 and 5), Gypsy (VdLTRE2/3/4)	nd
VdDvd-T5	ON	Tomato	2A	Copia (VdLTRE1), Gypsy (VdLTRE2/3/4)	Tc1/mariner (DAHLIAE1d), Activator (VdHAT1)
VdBB	ID	Potato	4A	Gypsy (VdLTRE2/3/4)	Tc1/mariner (DAHLIAE2), Activator (VdHAT2)
VdWM	TX	Cotton	2A	Gypsy (VdLTRE2/3/4)	nd
VdPH	CA	Pistachio	2A	Copia (VdLTRE1), Gypsy (VdLTRE2/3/4)	-
Vd115	Syria	Cotton	2B	Copia (VdLTRE1), Gypsy (VdLTRE2/3/4)	nd
VdS39	OH	Soil	4B	Copia (VdLTRE1), Gypsy (VdLTRE2/3/4)	Tc1/mariner (DAHLIAE2)
VdCW	WA	Cherry	4A/B	Copia (VdLTRE1), Gypsy (VdLTRE2/3/4)	Tc1/mariner (DAHLIAE2)
*V. albo-atrum*					
VaMs.102^a^	PA	Alfalfa	nd	Copia (VdLTRE1)	-
Va383-2	ON	Potato	nd	Copia (VdLTRE1)	Tc1/mariner (DAHLIAE2)
Va4ATC	ON	Potato	nd	Copia (VdLTRE1)	Tc1/mariner (DAHLIAE2)
VaV5591	CA	Cauliflower	nd	Copia (VdLTRE1), Gypsy (VdLTRE2/3/4)	-
Va462	MN	Potato	nd	nd	Tc1/mariner (DAHLIAE2)
VaChile1	Chile	Kiwi	nd	-	Tc1/mariner (DAHLIAE2)
VaPSU140	PA	*Ailanthus altissima*	nd	-	Tc1/mariner (DAHLIAE2)
Va48557	UK	Tomato	nd	-	Tc1/mariner (DAHLIAE2)
VaV481	UK	Hops	nd	-	Tc1/mariner (DAHLIAE2)
VaV104b	PEI	Potato	nd	-	nd
VaV4901	PEI	Potato	nd	-	nd

The presence of the indicated Class I and Class II transposable elements was determined by Southern hybridization or PCR amplification using gene-specific probes (amplicons) or primer pairs, respectively, as described in Materials and Methods and as shown in Additional file 8: Figure S6. ^a^Sequenced genomes; ^b^VCG, vegetative compatibility groups; -, not detected; nd, no data.

## Discussion

We originally set out to gain a better understanding of the nature and extent of genetic diversity in phytopathogenic *Verticillium* spp. and, to this end identified mobile elements in the sequenced *V. dahliae* and *V. albo-atrum* genomes, and explored their distribution in other strains of different origins. Our genome-wide search yielded complete retroelements and “cut-and-paste” DNA transposons, whose structure we characterized in detail. Among the VdLs.17 LTR sequences that we identified, the Ty1/Copia-like VdLTRE5 had the *GAG* and *POL* ORFs in an organization that is very rare [46], and uses a leaky stop codon for translation of the POL ORF that is downstream of the GAG ORF. VdLTRE5 did not have, downstream of the GAG stop codon, the conserved CARYYA sequence, which has been previously shown to be important for this stop codon read-through [24]. However, the Ty1/Copia-like element Tca2 of *Candida albicans* also does not possess this sequence and it has been proposed that the sequences responsible for stop codon read-through of Tca2 can be multiple, remote, and scattered throughout the element [47]. The same may therefore be true for VdLTRE5.

The VdLs.17 retrotransposons VdLTRE 2, 3, and 4 were identifiable as Gypsy elements in their having *GAG* and *POL* genes with the *POL* ORFs in a −1 frameshift orientation relative to that of the *GAG* ORF [46]. A notable feature differentiating these three TE families, which are predicted to encode almost identical polyproteins, was the difference in their LTR lengths. LTR elements contain critical *cis-*acting signals that define the element borders and act as transcriptional promoters [48], and we indeed found up to 5-fold differences in the number of the ESTs corresponding to each type of element.

Among the “cut-and-paste” TEs in VdLs.17, the Tc1/mariner superfamily, which derives its name from the founder transposons Tc1 of *Caenorhabditis elegans* and mariner of *Drosophila mauritiana*[8], was predominant. All 29 full-length DAHLIAE elements were approx. 2 Kb in size and comprised single intronless DDE_1-encoding transposases flanked by short (41–102 bp) TIRs. The DDE_1 transposases act generally as dimers or oligomers and harbor functional domains mediating protein-protein interaction, DNA-binding, -cleavage and -joining activities [49]. We predicted the presence of two types of N-terminal DNA-binding domains, HTH_psq and HTH_Tnp_Tc5, which are involved in the recognition and interaction with the TIRs [50]. The DDE_1 domain, first identified in bacterial transposases and retroviral integrases, is characterized by conservation of three aspartate (D) residues or two, non-contiguous (D) and one glutamate (E) residue, a catalytic triad that forms a pocket able to bind two divalent metal ions, mostly likely Mg2+, that are necessary for transposition [51]. The DDE signature has been detected in all eukaryotic “cut-and-paste” transposase superfamilies, indicating their common evolutionary origin [52]. In the DAHLIAE transposases, the three aspartate residues are separated by 110–112 and 35 amino acids, respectively (D110-112D35D).

*In vitro* and *in vivo* trans-kingdom assays have previously demonstrated that, while host factors are not needed for transposition, intact transposase and TIRs are required for the initiation and completion of the process [53-56]. Tc1/mariner family transposon termini comprise at least three types of functional sequences involved in transposition: the 4–7 nucleotide TE cleavage sites at the outer extremities of the TIRs, the DRs within the TIRs, which are the transposase binding sites, and the UTRs, between the TIRs and ORFs, which act as enhancers of transposition efficiency [49,57-59]. Transposons of different families generally differ in their terminus structure and length, as well as in the transposase domain architecture. Each terminus/transposase combination appears to mediate a slightly different version of the “cut-and-paste” mobilization mechanism, ensuring transposition specificity [49]. In VdLs.17, while DAHLIAE3 starts with the unique cleavage motif CCCG and does not possess recognizable repeated sequences in the TIRs like those of Tc1/mariner TEs of other ascomycete fungi [60], DAHLIAE 1 and 2 start with the sequence ACGT-, and their TIRs contain two or three DRs. In particular, DAHLIAE2 carries three internal repetitions of a 17 bp-sequence at the 5’ terminus, and two at the 3’ terminus. The DAHLIAE ORFs do not overlap with the TIR sequences and are flanked by asymmetric UTRs that vary in length from 33 to 125 bp, according to the TE family type. The VdLs.17 Activator and Mutator elements are highly degenerate (VdHATs) or with limited sequence similarities (VdMULEs). Most of the elements appeared to be of the non-autonomous type due to mutations that disrupted TIR sequences and/or resulted in incomplete transposases. Although these elements are probably unable to transpose and are fossils, we were able to identify corresponding ESTs for some of them. These sequences may therefore still play the important role of repressing transposition of the complete elements of the same family through transposase dilution or through a negative dominant repression by the truncated transposases.

Domestication is the process by which TE functional domains are incorporated into functional host proteins [5]. In VdLs.17 we found an insertion of a fragment of a Mutator element within a nitrilase gene. The fused sequence is predicted to generate a protein carrying in-frame nitrilase and MULE domains. Although we did not find corresponding ESTs, we cannot rule out the possibility that this new protein is functional.

The EST data and expression analysis under heat stress further showed that several of the full length Class I and II TEs, which are predicted to code for complete transposition-mediating enzymes, are still transcriptionally active and differentially responsive to different stimuli.

In general, our phylogenetic analysis of both Class I and II TEs mirrored the relationships among the fungal species that were defined by the fungal genome initiative on the basis of whole genome comparative studies. The evolutionary ancestors of the Copia, DAHLIAE, VdHAT and VdMULE TEs apparently evolved into different groups before insertion into the VdLs.17 genome. While high bootstrap values supported the monophyletic evolution of the *V. dahliae* Tc1/mariner and Mule elements, the Copia VdLTREs 1 and 5 and the VdHATs fell into distinct lineages. The three Gypsy families diversified after introgression into VdLs17 genome. Also, the Tc1/mariner family DAHLIAE1 underwent a recent expansion in VdLs.17, generating five VdLs.17-specific subfamilies. These subfamilies differed in sequence and length of their termini, and of their ORF sequences. DAHLIAE1 a, b and d all putatively coded for intact transposases and were present in multiple, almost identical copies, comprising 74% of the total DNA TEs in VdLs.17. In fungi, the selective amplification of transposon subfamilies, such as we have detected for the DAHLIAE1 elements, has been associated to events of horizontal gene transfer [18], however no definitive mechanism has yet been proven.

It has been proposed that TEs may enhance recombination to cause genetic variation, giving populations the flexibility to adapt, a phenomenon which could be especially important for species that do not have a sexual phase [18]. TE clustering such as we observed in the VdLs.17 LS regions has been found in the genomes of other phytophathogenic fungi. In *M. oryzae*, for example, both Class I and II transposable elements are clustered within three regions of chromosome seven, characterized by a high rate of duplication and evolution [13]. Similarly, TEs were also found to cluster in regions undergoing rapid reorganization in the genome of the plant vascular pathogen *F. oxysporum*[61]. It has been hypothesized that these types of clusters are important for the generation and evolution of new genes [13], and it has been proposed that the transposon-rich LS regions in *V. dahliae* may impart a degree of genetic flexibility in the species, and allow rapid adaptation to new host niches [2]. The mechanism(s) by which TE clusters are generated in fungal genomes has not been elucidated yet. While the clustering could simply be the passive result of selection against TE incorporation into gene-rich zones of the genome, it alternatively could result from an active process related to TE function. Many TEs do selectively integrate into specific sequences [62,63], which could lead to biased TE distribution. Such selectivity has, for example, been observed for *S. cerevisiae*, in which Ty3 LTR elements are most often found to be integrated into upstream regions of genes transcribed by RNA polymerase III [64]. The parallel, non-random clustering of the Class I and II *V. dahliae* elements suggests that there may be synergistic interactions among different types of elements, and selective pressure in *V. dahliae* for TE clustering, which in turn may be important for generating the genomic diversity necessary for niche adaptation and host range expansion. Interestingly, among the 354 predicted genes encoded within the LS regions, there were no “housekeeping” genes [2]. Rather, the predicted genes encoded proteins of potential importance in pathogenicity, including bZIP transcription factors, ferric reductases, phospholipases, and other genes predicted to play a role in response to stress [2]. Moreover, clusters or pairs of genes were clearly duplicated in these LS regions ([2] and this study). It is unclear if such duplications are the direct result of TE activity, and more studies are required to investigate the role(s) of these putative pathogenicity factors. However, the presence of solo-LTR sequences does suggest that recombination between the repeated sequences of the LTR elements could be a contributing factor in the reorganization of *Verticillium dahliae* genome.

Since TEs can have a large effect on the genomes of their hosts, causing gene deletion and duplication as well as chromosomal rearrangements, host fitness could be adversely affected if TE-induced transposition and recombination events disrupted or altered function of essential genes. However, some filamentous fungi have a unique tool, known as repeat-induced point mutation (RIP), to deal with repetitive sequences such as TEs. RIP, a process first described in *Neurospora crassa*[22], was found to occur during the sexual cycle, and to subject duplicated sequences of >400 base pairs to irreversible CG to TA transition point mutations. Although RIP is known to only occur during the fungal sexual cycle, there are several examples in asexual fungi where TEs with RIP-like mutations have been identified [23,65,66]. The identification of RIP-like mutations in some VdLTRE2/3/4 sequences [2] indicated that, at some point in *Verticillium* spp. evolution, a RIP-like process operated to protect the genome from infiltration by TEs. The presence in *V. dahliae* and *V. albo-atrum* of identifiable RID-like protein orthologs, which are known to be a part of the RIP machinery in *N. crassa*[67], suggests that these fungi may still possess the capacity for RIP. Interestingly, in VdLs.17, RIP mutations affected members of the Gypsy but not Copia superfamilies of retrotransposons, indicating either a differential susceptibility to RIP by different types of TEs or introgression by horizontal transfer of already RIPed sequences [2].

The TE content of different organisms is variable, sometimes accounting for as much as 60–80% of the genome, as in the case of cereals. More recently, mobile elements have been found to account for about half of the genome size also in the phytopathogenic oomycete *Phytophthora infestans* and the truffle fungus *Tuber melanosporum*[12]. Noticeably, a high degree of variability in the number and types of transposons has been documented even in closely related species. For instance, the TE content of the fungal plant pathogens *F. oxysporum* and *F. graminearum* differs by a two-fold order of magnitude, 4% and 0.03%, respectively [68]. The observed TE spatial-temporal fluctuations appear to be TE-family dependent and governed by multiple mechanisms including elimination by ectopic recombination, extinction by genetic drift, reintroduction by horizontal transfer, and environmental stress-driven expansion (reviewed by Hua-Van et al. [12]). Despite the high level of identity (97%) and synteny identified between the genomes of the recently sequenced Vd and Va isolates, the *V. albo-atrum* genome assembly was distinct in containing far less repetitive DNA than did that of *V. dahliae*[2]. In particular, VaMs.102 lacked the highly repetitive LS regions present in VdLs.17, and contained neither full-length nor defective VdLTRE5 Copia or “cut-and-paste” DNA elements. This absence seems simply to reflect the isolate of *V. albo-atrum* sequenced rather than a general feature of the species. In fact, 66% of the other Va isolates we surveyed for the presence of Vd.Ls17 TE-like sequences were positive for the Tc1/mariner DAHLIAE2.

Extensive studies conducted on natural populations of other organisms such as *Drosophila* and different plant species including *Arabidopsis*, barley, maize, rice and wheat have clearly demonstrated that transposon dynamics plays a central role also in generating intraspecific variability [69-73]. Our findings have shown that the Copia-like VdLTRE1 and the Gypsy-like retrotransposons are almost ubiquitous in *V. dahliae*, whereas the Copia-like VdLTRE5 and most of the “cut-and-paste” DNA TEs appear to have a much more limited distribution. Although lack of detection of VdLs.17-like TEs could indeed reflect total absence of mobile elements, those *Verticillium* genomes without such elements may either contain related sequences that have diverged to a degree that prevented detection under the conditions used in this study (see Materials and Methods), or may simply harbor their own distinct arrays of transposable elements.

## Conclusions

The identification and characterization of Class I and II TEs of *Verticillium dahliae* (VdLs.17) has allowed further exploration of the genetic diversity existing among the phytopathogenic *Verticillium* spp., and raised intriguing questions about the role(s) that TEs may have in shaping intra- and inter-specific evolution in this fungal genus. The discovery of chromosome location “hot spots” for TE insertion, as well as a “patchy” distribution among isolates of some of the TEs offers tantalizing evidence that TEs may play an important role in modulating genome architecture to allow *Verticillium* fungi to evolve quickly and adapt to their hosts, as has been suggested for other plant pathogens [17,74]. *V. dahliae* and *V. albo-atrum* were previously perceived, on the basis of vegetative compatibility and pathogenicity analyses, to have rather low genetic diversity. However, the results of molecular analyses carried out over the past two decades suggest that the species do in fact exhibit a high level of genetic variation and genome plasticity [3,75,76]. The analyses presented herein showed a broad range of different combinations of TE types in the different fungal strains, further supporting a major contribution of the transposable elements to the diversification of *Verticillium* spp. genomes.

## Methods

### Bioinformatics and characterization of *Verticillium* Class I and II transposons

The genome sequences of the fungal strains *V. dahliae* VdLs.17 and *V. albo-atrum* VaMs.102, and the associated expressed sequence tag (EST) information for strain VdLs.17 are available at http://www.broadinstitute.org/annotation/genome/verticillium_dahliae/MultiHome.html (Broad Institute), and served as the primary source for the TEs identified in this study. Virulence data and other information on these strains were previously published [77,78]. To identify repetitive sequences in the *V. dahliae* VdLS.17 and *V. albo-atrum* VaMs.102 genomes, sequences were searched using Cross_match [79], and filtering for alignments longer than 200 bp with greater than 60% sequence similarity, as described in Klosterman et al. [2]. Class I and II transposon sequences were initially identified by searching among the repetitive sequences for putative transposon elements using computational predictions based on BLASTX analysis. For identification of the Class I elements, sequences from the above collection of putative transposon loci were manually inspected using the DNASTAR v. 6 software suite (DNASTAR Inc., Madison WI). Direct repeats flanking putative *GAG* and *POL* ORFs, a hallmark of Class I LTR elements, were first identified using the DNAstar program GeneQuest 6.1. Putative LTR and LINE-like element sequences were then aligned with ClustalW to identify type elements of the LTR-containing Ty3/Gypsy, Ty1/Copia, and LINE-like class TEs. Conserved domain analysis of predicted open reading frames (ORFs) encoded by the putative LTR elements was conducted using the NCBI conserved-domain database search tool [80,81], and the GenomeNet MOTIF search at http://motif.genome.jp. For the identification of the Class II “cut-and-paste” elements, sequences from the initial collection of putative transposon loci were manually inspected with homology-based methods including BLASTN searches of VdLs.17 and VaMs.102 genomes and NCBI databases. VdLs.17 genomic regions flanking the regions of homologies (3 to 6 Kb total) were then self-aligned using BLASTN align program to identify TIRs. Identification of DNA transposons was also based on use of the feature search tools available at the *Verticillium* group Database, in particular the feature type HMMR and the text search for DDE and hAT. We also searched the sequenced *Verticillium* genomes with complete or individual conserved domain aa sequences of well-characterized transposases of other closely related fungi. Nucleotide sequences of the identified full-length and defective VdLs.17 TEs were conceptually translated using TRANSLATE in ExPASy, and used in BLASTX searches to identify transposase conserved domains and the closest homologous sequences from other organisms.

### Phylogenetic analyses

Nucleotide and amino acid sequences were aligned using the programs CLUSTALW and MUSCLE. Sequences were collapsed into haplotypes by manually removing INDELS using the multiple alignment editor program Jalview. Maximum-likelihood distance trees were inferred by using PhyML 3.0, and selecting the substitution models and at least 1000 bootstrap replications. Trees were graphically represented using the program TreeView. For the separation of the DAHLAIE1 elements into the subfamilies a to e, we used the TE full-length nucleotide sequences (inclusive of TIRs). While the analysis of the Tc1/mariner transposases was based on complete protein sequences, the low level of conservation among VdHATs and VdMULEs allowed for an evaluation of relatedness based on the conserved hAT and MULE domains only. Conserved amino acid positions within each transposase functional domain were identified by visualizing their sequence alignments using the program MAFFT in Jalview.

### Fungal strains and growth conditions

With the exception of V. *albo-atrum* isolates V104b and V4901 (provided by Dr. F. Daayf, University of Manitoba, Winnipeg MB), all *Verticillium* isolates used in this study (Table 3) are from culture collections of K.F.D, M.d.M.J. and P.V. Isolates were stored long-term at −20°C as filter paper stock. Cultures were routinely grown at 24°C on complete medium (CM) agar and in CM broth [82].

### Nucleic acid isolation manipulations

Fungal genomic DNA was isolated from *Verticillium* spores using a glass-bead breakage method as described previously [82]. For DNA blot hybridization, restriction enzyme-digested genomic DNA was electrophoretically size-fractionated through agarose gels, transferred by capillary blotting to positively charged nylon membranes (Roche Diagnostics, Indianapolis, IN), and fixed to the membrane by UV cross-linking. High stringency hybridizations (65°C), and chemiluminescent detection of DIG-labeled hybrids were done as described previously [83]. Hybridization probes were synthesized from *V. dahliae* VdLs.17 gDNA by the incorporation of DIG-labeled dUTP into PCR amplification products. Amplification reactions contained Platinum Taq polymerase (Invitrogen, Canda Inc., Burlington, ON), DIG Labeling Mix (Roche Diagnostics, Indianapolis, IN), DNA, and forward and reverse primer pairs indicated in Additional file 9: Table S3. Reaction conditions involved an initial 2 min denaturation at 94°C, followed by 30 amplification cycles: 94°C for 45 sec, 65°C for 45 sec, 72°C for 60 sec, and a final 5 min extension at 72°C. PCR-based genomic survey for the presence of specific TEs in Vd and Va strains was performed using 20 ng of genomic DNA in a 25 μL reaction mixture containing 1.5 mM MgCl_2_, 100 mM dNTPs, 100 pmol of each TE-specific primer and 1 unit of Taq DNA polymersase (Promega). As controls of DNA equal loading and amplification specificity, we included in the experiments amplification of a VdLs.17 actin gene (VDAG_08445) and no-DNA samples. The sequences of the primers are listed in Additional file 9: Table S3.

### Transcriptional analysis

The analysis of the expression of selected transposase genes was performed by semi-quantitative reverse transcription PCR (RT-PCR) experiments. Total RNA was extracted from VdLs.17 grown under control conditions (25°C) or exposed to heat (42°C) for 40 minutes using RNeasy Plant Mini Kit (QIAGEN). An in-column DNase step was added according to the manufacturer’s instructions. First strand cDNA was synthetized using 500 ng total RNA, oligo (dT) and TaqMan Reverse Transcription kit (Applied Biosystems). PCR amplification were done using one 1 ul cDNA in 20 μl reactions containing 1.5 mM MgCl_2_, 100 mM dNTPs, 100 pmol of each transposase-specific primer, 1 unit Taq DNA polymersase (Promega), and 30 amplification cycles. As controls of cDNA equal loading and amplification specificity, we included in the experiments amplification of cDNA corresponding to a VdLs.17 actin gene (VDAG_08445) and no-cDNA samples. The primers were designed on the basis of EST sequence information publicly available at the *Verticillium* Group Database/Fungal Genome Initiative/Broad Institute of MIT and Harvard (http://www.broadinstitute.org). Primer sequences are listed in the Additional file 9: Table S3.

### Statistical analyses of transposon clustering in the genome of *V. dahliae*

Odds ratio analyses [37] of transposons in the LS regions of the genome of isolate VdLs.17 of *V. dahliae* versus those encoded in the core genomic sequences of VdLs.17 were carried as described [2]. The only exception was that each Class I or II transposon type was parsed into numbers within LS regions or numbers in non-LS regions, and then grouped with the total numbers of genes within the LS regions or non LS regions for analyses. A total of 354 LS genes were used in the analyses and the total number of genes, determined by the Broad Institute annotation pipeline, was 10,535.

### Analyses of repeat-induced point mutation

Sequences corresponding to each of the transposon families were identified by a BLASTN search of the *V. dahliae* genome (E value cutoff < 1E^-5^) with the type elements. Sequences with a length of at least 500 bp were then retrieved and aligned using MUSCLE [84], and analyzed using the RIPCAL software [44].

## Competing interests

The authors declare no competing financial interests.

## Author’s contributions

S.G.A. and X.T. identified and characterized retrotransposons and DNA transposons, respectively; K.P. and S.G.A. contributed to the survey of Vd and VA isolates for the presence of TEs, and K.P. to the RT-PCR expression studies; M.d.M.J. contributed to the survey of Vd and VA for the presence of DNA TEs; S.J.K. and L.J.M. contributed to the analysis of the TE distribution within the VdLs.17 sequenced genome; L.J.M. identified the candidate transposons based on bioinformatic analysis; K.F.D. and P.V. conceived the study, and contributed to the transposon discovery and characterization. All authors contributed to the writing and critical revision of the manuscript, and read and approved the final manuscript.

## Supplementary Material

Additonal file 1 **Figure S1. Alignments of the conserved domains of VdLs.17 Tc1/mariner-like DAHLIAE transposases.** The amino acid alignments of the conserved domains DDE_1 (A), HTH_psq (B) and HTH_Tnp_Tc5 (C), which characterize the transposases of the Tc1/mariner element DAHLIAE are shown. The alignments were generated using CLUSTALW and the amino acid conservation visualized using Jalview tools. The red boxes indicate the positions of the aspartic acid triad that characterize the endonuclease DDE_1 motif. The alignments include sequences from other fungi that are the best matches of the DAHLIAE transposases (listed in Table 1). Click here for file

Additonal file 2 **Figure S2. Terminal inverted repeats (TIRs) of the Class II elements identified in the VdLs.17 genome.** The TIR nucleotide sequences of the Tc1/mariner-like elements DAHLAIE 1 and 2 (A), the Activator elements VdHAT 1 and 2 (B) and of the Mutator elements VdMULE 1 to 3 are shown. The internal direct duplications are underlined. The asterisks indicate mismatches. Click here for file

Additonal file 3 **Figure S3. Alignment of VdLs.17 hAT and MULE transposase domains.** The amino acid alignments used for the phylogenetic analysis of the VdHAT (A) and VdMULE (B) elements are shown. The alignments were generated using CLUSTALW, all gaps were removed manually and the amino acid conservation visualized using Jalview tools. Click here for file

Additional file 4 Table S1. Activator-and Mutator- like transposons identified in the VdLs.17 genome. Click here for file

Additional file 5 Table S2. Odd ratio analysis of Class I and Class II transposon domain encoded in the genome of VdLs.17. Click here for file

Additonal file 6 **Figure S4. Analysis of the expression of VdLs. 17 Class II transposases in response to heat stress.** The transcription of the indicated Class II transposases was analyzed by RT-PCR experiments using cDNA synthetized from total RNA extracted from VdLs.17 grown at 25°C or exposed to heat stress (42°C for 45 minutes). The primers used to amplify the transposase ORFs were designed on the basis of the sequence of the corresponding ESTs publicly available at the *Verticillium* Group Database (http://www.broadinstitute.org/annotation/genome/verticillium_dahliae/MultiHome.html) (Primer sequences are listed in Supplemental Table 3). Click here for file

Additonal file 7 **Figure S5. RIPCAL analysis of VdLs.17 Class II TEs.** Search of the indicated DNA transposon sequences for evidence of RIP mutations by RIPCAL analyses did not show any clear bias in the type of mutations observed. Analysis of VdLTRE2-like sequences, in which a bias for CA to TA transition mutation was previously identified [2] was included in our study as a positive control of RIPing. Click here for file

Additonal file 8 **Figure S6. Distribution of Class I and II elements identified in the Vd.Ls17 genome in other phytopathogenic*Verticillium*spp. isolates.** A) Southern hybridization analysis of the indicated Vd and Va fungal isolates for the presence of retrotransposons. Genomic DNA was digested with *Eco*RI (top panel), *Bgl*II (middle panel), or *Sac*I (bottom panel), and blots hybridized with DIG-labelled probes corresponding to VdLTRE1, VdLTRE2/3/4, or VdLTRE5, respectively. Sizes of DIG-labelled molecular weight markers (Kb) are indicated to the right of the images; B) PCR amplifications of retrotransposons in the indicated Vd and Va isolates, using VdLTRE1, VdLTRE2 and VdLTRE5 primer pairs. Amplification of actin was used as control (not shown); FSDW, no-template reaction; C) PCR amplification of DAHLIAE, VdHAT and VdMULE elements was obtained by using as a template genomic DNA (20 ng) of the indicated Vd and Va isolates; D) To verify specificity of the bands of different size obtained from amplification of DAHLIAE 1d, PCR products were blotted onto nylon membranes and hybridized with DIG-labelled specific probes. Details of the isolates used in the survey are provided in Table 3. Sequences of the primers used in the survey are listed in Supplemental Table 3. Click here for file

Additional file 9 Table S3. List of PCR amplification primers used in this study. Click here for file
