# Supplementary figures and images for: Transposable elements in phytopathogenic Verticillium spp.: insights into genome evolution and inter- and intra-specific diversification
Source: BMC Genomics. 2012 Jul 16;13:314. doi: 10.1186/1471-2164-13-314 (PMC3441728; doi:10.1186/1471-2164-13-314)

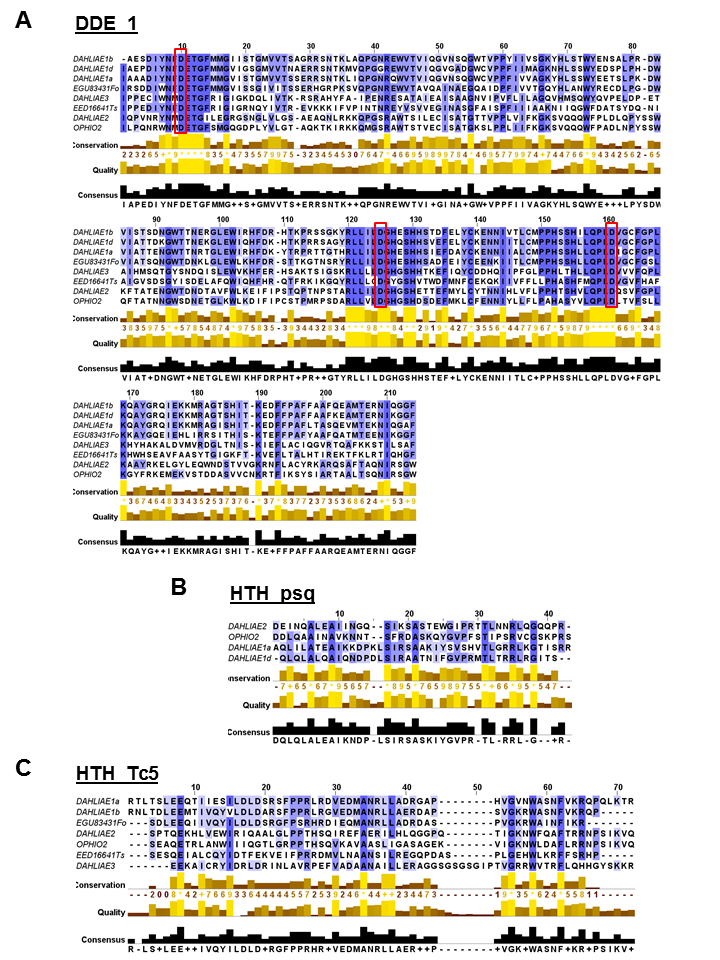


**Supplemental Figure 1**

**Amyotte *et al*., 2012**

Supplement: Additonal file 1 — Figure S1. Alignments of the conserved domains of VdLs.17 Tc1/mariner-like DAHLIAE transposases. The amino acid alignments of the conserved domains DDE_1 (A), HTH_psq (B) and HTH_Tnp_Tc5 (C), which characterize the transposases of the Tc1/mariner element DAHLIAE are shown. The alignments were generated using CLUSTALW and the amino acid conservation visualized using Jalview tools. The red boxes indicate the positions of the aspartic acid triad that characterize the endonuclease DDE_1 motif. The alignments include sequences from other fungi that are the best matches of the DAHLIAE transposases (listed in Table 1). [file 1471-2164-13-314-S1.docx]

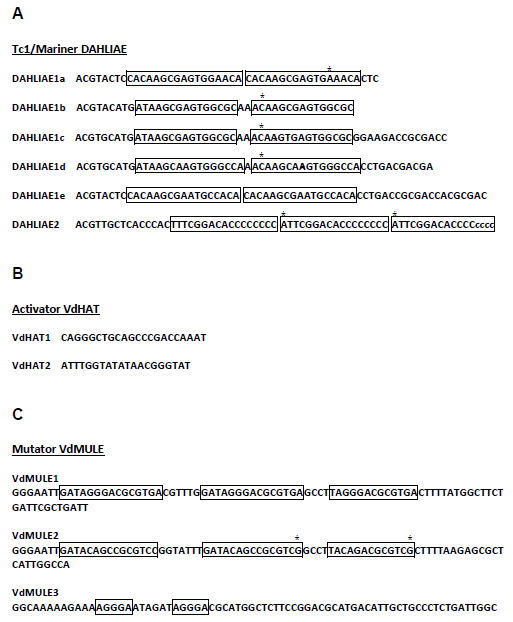


**Supplemental Figure 2**

**Amyotte *et al*., 2012**

Supplement: Additonal file 2 — Figure S2. Terminal inverted repeats (TIRs) of the Class II elements identified in the VdLs.17 genome. The TIR nucleotide sequences of the Tc1/mariner-like elements DAHLAIE 1 and 2 (A), the Activator elements VdHAT 1 and 2 (B) and of the Mutator elements VdMULE 1 to 3 are shown. The internal direct duplications are underlined. The asterisks indicate mismatches. [file 1471-2164-13-314-S2.docx]

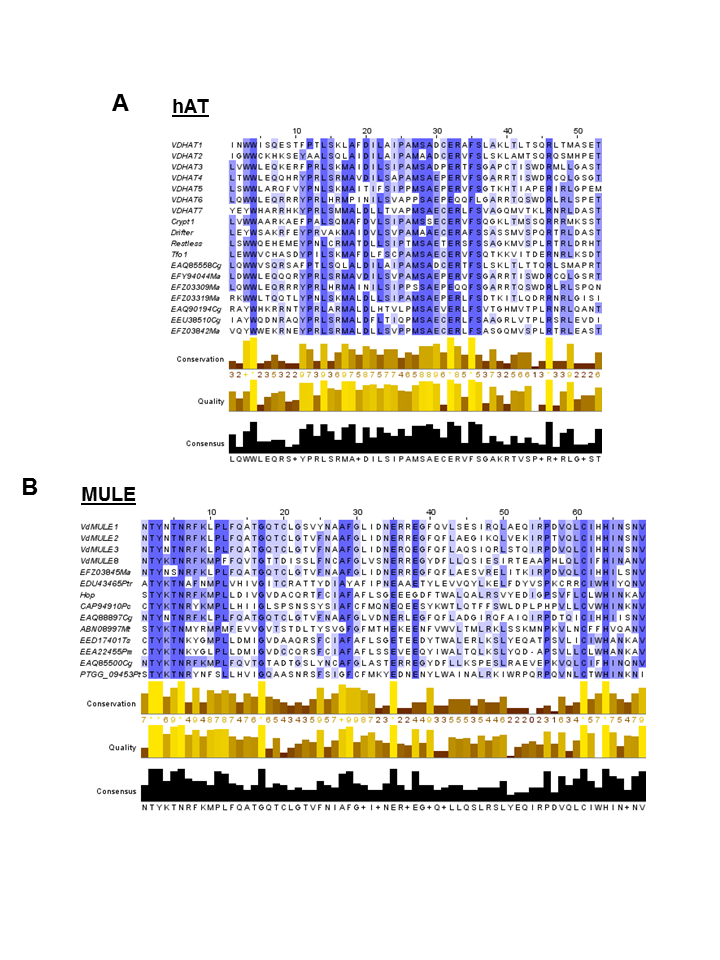
**Supplemental Figure 3**

**Amyotte *et al*., 2012**

Supplement: Additonal file 3 — Figure S3. Alignment of VdLs.17 hAT and MULE transposase domains. The amino acid alignments used for the phylogenetic analysis of the VdHAT (A) and VdMULE (B) elements are shown. The alignments were generated using CLUSTALW, all gaps were removed manually and the amino acid conservation visualized using Jalview tools. [file 1471-2164-13-314-S3.docx]

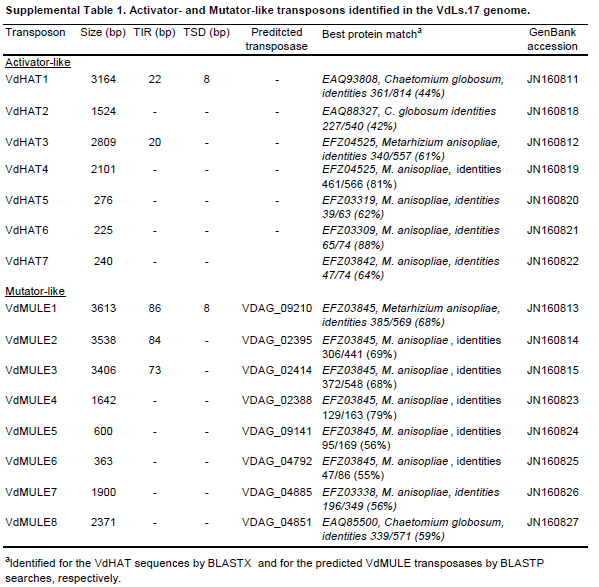


**Supplemental Table 1**

**Amyotte *et al*., 2012**

Supplement: Additional file 4 — Table S1. Activator-and Mutator- like transposons identified in the VdLs.17 genome. [file 1471-2164-13-314-S4.docx]

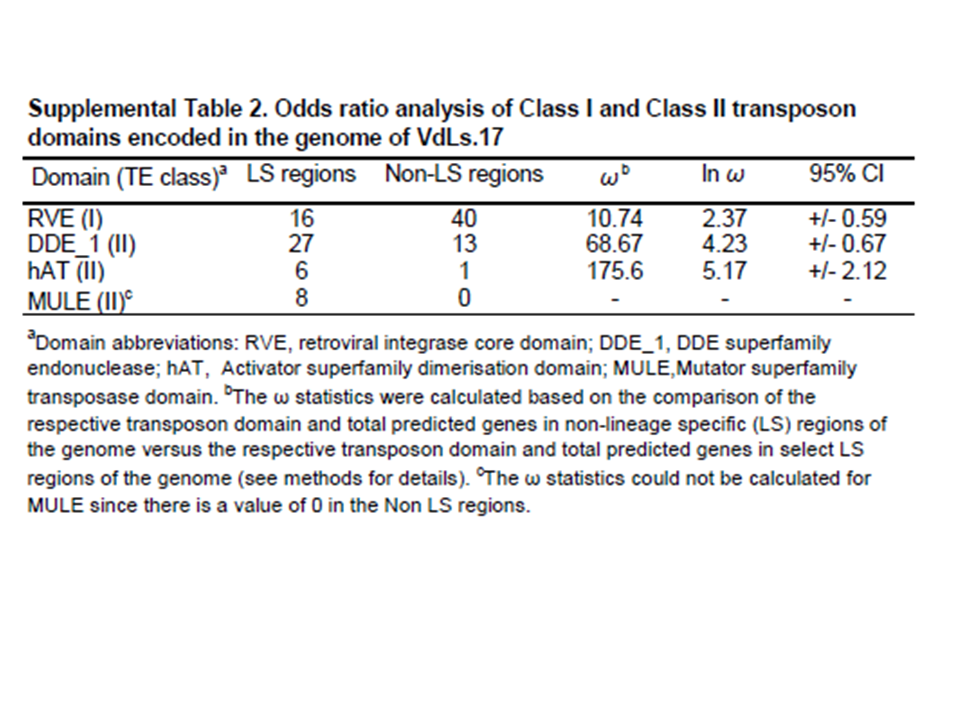
**Supplemental Table 2**

**Amyotte *et al*., 2012**

Supplement: Additional file 5 — Table S2. Odd ratio analysis of Class I and Class II transposon domain encoded in the genome of VdLs.17. [file 1471-2164-13-314-S5.docx]

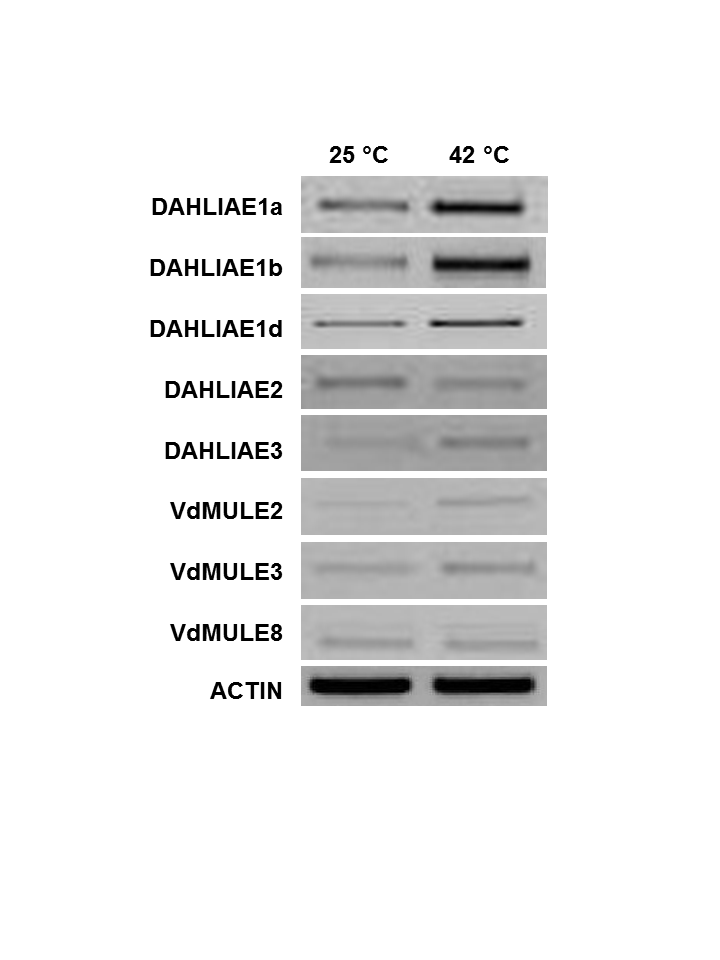


**Supplemental Figure 4**

**Amyotte *et al*., 2012**

Supplement: Additonal file 6 — Figure S4. Analysis of the expression of VdLs. 17 Class II transposases in response to heat stress. The transcription of the indicated Class II transposases was analyzed by RT-PCR experiments using cDNA synthetized from total RNA extracted from VdLs.17 grown at 25°C or exposed to heat stress (42°C for 45 minutes). The primers used to amplify the transposase ORFs were designed on the basis of the sequence of the corresponding ESTs publicly available at the Verticillium Group Database (http://www.broadinstitute.org/annotation/genome/verticillium_dahliae/MultiHome.html) (Primer sequences are listed in Supplemental Table 3). [file 1471-2164-13-314-S6.docx]

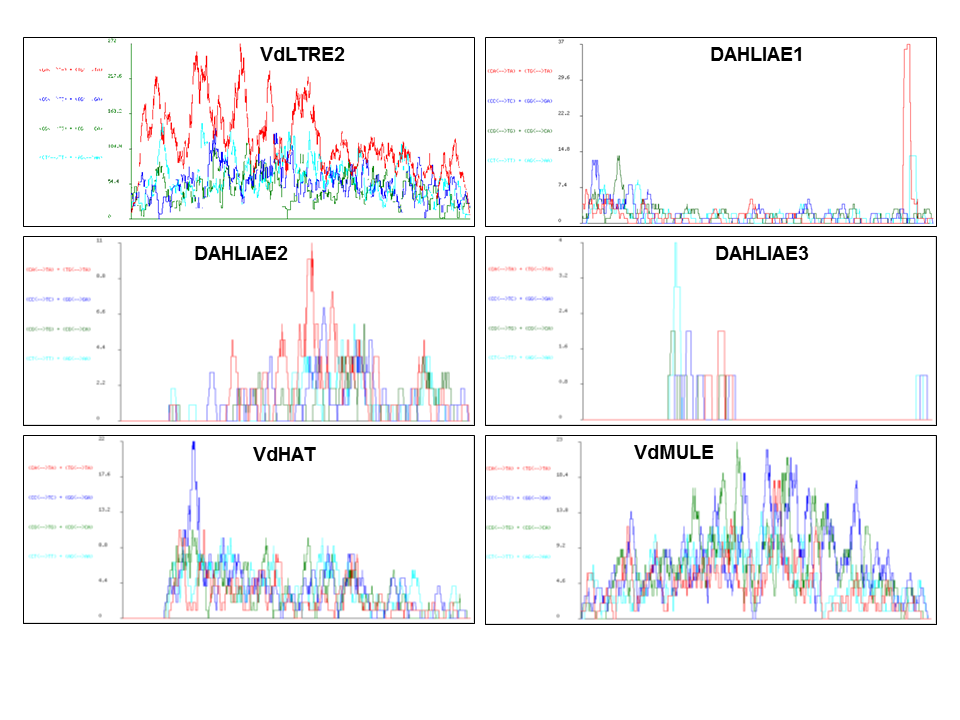


**Supplemental Figure 5**

**Amyotte *et al*., 2012**

Supplement: Additonal file 7 — Figure S5. RIPCAL analysis of VdLs.17 Class II TEs. Search of the indicated DNA transposon sequences for evidence of RIP mutations by RIPCAL analyses did not show any clear bias in the type of mutations observed. Analysis of VdLTRE2-like sequences, in which a bias for CA to TA transition mutation was previously identified [2] was included in our study as a positive control of RIPing. [file 1471-2164-13-314-S7.docx]

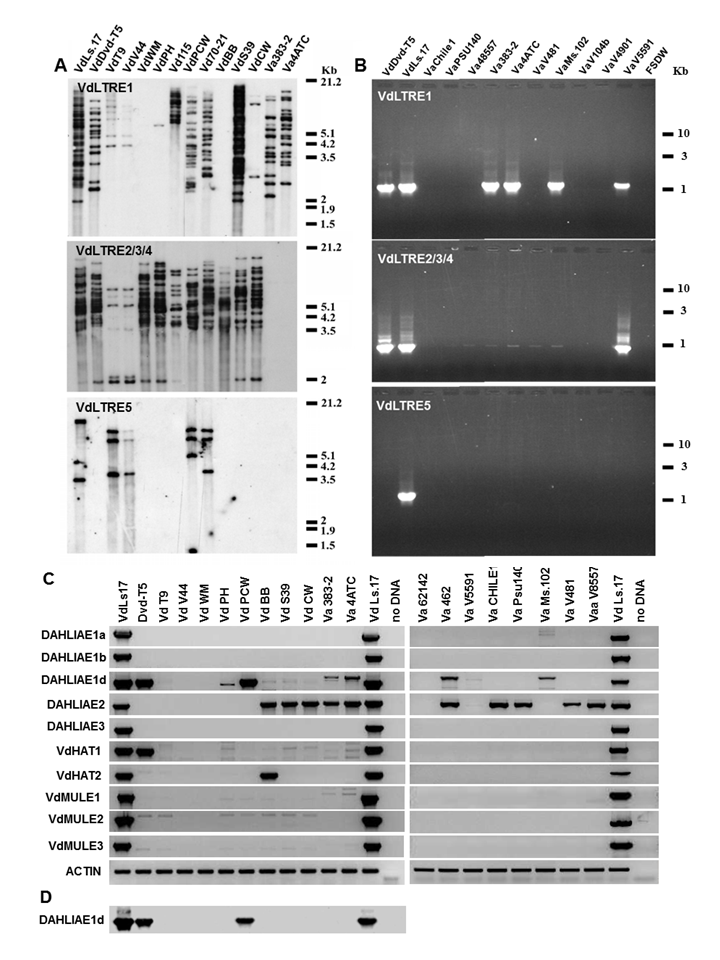


**Supplemental Figure 6**

**Amyotte *et al*., 2012**

Supplement: Additonal file 8 — Figure S6. Distribution of Class I and II elements identified in the Vd.Ls17 genome in other phytopathogenicVerticilliumspp. isolates. A) Southern hybridization analysis of the indicated Vd and Va fungal isolates for the presence of retrotransposons. Genomic DNA was digested with EcoRI (top panel), BglII (middle panel), or SacI (bottom panel), and blots hybridized with DIG-labelled probes corresponding to VdLTRE1, VdLTRE2/3/4, or VdLTRE5, respectively. Sizes of DIG-labelled molecular weight markers (Kb) are indicated to the right of the images; B) PCR amplifications of retrotransposons in the indicated Vd and Va isolates, using VdLTRE1, VdLTRE2 and VdLTRE5 primer pairs. Amplification of actin was used as control (not shown); FSDW, no-template reaction; C) PCR amplification of DAHLIAE, VdHAT and VdMULE elements was obtained by using as a template genomic DNA (20 ng) of the indicated Vd and Va isolates; D) To verify specificity of the bands of different size obtained from amplification of DAHLIAE 1d, PCR products were blotted onto nylon membranes and hybridized with DIG-labelled specific probes. Details of the isolates used in the survey are provided in Table 3. Sequences of the primers used in the survey are listed in Supplemental Table 3. [file 1471-2164-13-314-S8.docx]

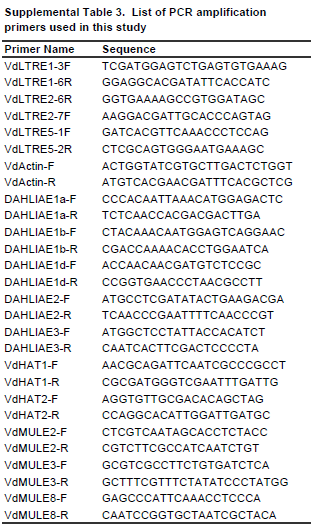


**Supplemental Table 3**

**Amyotte *et al*., 2012**

Supplement: Additional file 9 — Table S3. List of PCR amplification primers used in this study. [file 1471-2164-13-314-S9.docx]
